# Supplementary material for: Contraceptive use by number of living children in Ghana: Evidence from the 2017 maternal health survey
Source: PLoS One. 2023 Dec 14;18(12):e0295815. doi: 10.1371/journal.pone.0295815 (PMC10721096; doi:10.1371/journal.pone.0295815)

PLOS ONE

Contraceptive use by number of living children in Ghana: evidence from the 2017 maternal health survey

--Manuscript Draft--

**Manuscript Number:**	PONE-D-23-19366
**Article Type:**	Research Article
**Full Title:**	Contraceptive use by number of living children in Ghana: evidence from the 2017 maternal health survey
**Short Title:**	Contraceptive use by number of living children in Ghana
**Corresponding Author:**	Sarah Compton, PhD, MPH<br>University of Michgian<br>Ann Arbor, MI UNITED STATES
**Keywords:**	contraceptive choice; Ghana; highly effective contraception; parity
**Abstract:**	Background: There is a significant literature describing the link between parity and contraceptive use. However, there is limited knowledge about the disaggregation by parity of the type of contraceptives. In this study, we describe the use of contraceptives by parity among women of reproductive age in Ghana, focusing on use of highly effective methods (injection, pill, intrauterine device, implant, and sterilization).<br>Methods: Using the 2017 Ghana Maternal Health Survey, a nationally-representative cross-sectional household survey, we describe contraceptive method use by number of living children among sexually active women of reproductive age. We then estimated predictors of use of highly effective contraception in a multilevel logistic regression model.<br>Results: Most women in this survey are not using any method of contraception, although this varies by whether or not they have begun childbearing. Contraceptive method use varies by number of living children. Before having children, natural (periodic abstinence and withdrawal) and episodic (condoms) methods dominate. Once a woman has one living child, method preference changes to injectables and implants. Factors associated with using a highly effective method of contraception are: having >3 children, being in a relationship, having had an abortion, being younger than age 30, and having had sexual intercourse within days of answering the survey.<br>Conclusion: In this analysis, the number of living children a woman has, her age, and timing of last intercourse are the most significant predictors of using a highly effective method of contraception. However, the majority of participants in this study report not using any method of contraception to avoid unwanted pregnancies. Future research that attempts to unpack the disconnect between not wanting to become pregnant and not using contraception is warranted.
**Order of Authors:**	Sarah Compton, PhD, MPH
	Emmanuel Nakua, PhD, MS
	Cheryl Moyer, PhD, MPH
	Veronica Dzomeku, PhD
	Emily Treleaven, PhD, MPH
	Easmon Otupiri, PhD
	Jody Lori, PhD, CNM
**Additional Information:**	
**Question**	**Response**
**Financial Disclosure**<br><br>Enter a financial disclosure statement that describes the sources of funding for the	The author(s) received no specific funding for this work.

Powered by Editorial Manager® and ProduXion Manager® from Aries Systems Corporation

work included in this submission. Review the submission guidelines for detailed requirements. View published research articles from *PLOS ONE* for specific examples.<br><br>This statement is required for submission and **will appear in the published article** if the submission is accepted. Please make sure it is accurate.<br><br>**Unfunded studies**<br>Enter: *The author(s) received no specific funding for this work.*<br><br>**Funded studies**<br>Enter a statement with the following details:<br>• Initials of the authors who received each award<br>• Grant numbers awarded to each author<br>• The full name of each funder<br>• URL of each funder website<br>• Did the sponsors or funders play any role in the study design, data collection and analysis, decision to publish, or preparation of the manuscript?<br>• **NO** - Include this sentence at the end of your statement: *The funders had no role in study design, data collection and analysis, decision to publish, or preparation of the manuscript.*<br>• **YES** - Specify the role(s) played.<br><br>* typeset	
**Competing Interests**<br><br>Use the instructions below to enter a competing interest statement for this submission. On behalf of all authors, disclose any competing interests that could be perceived to bias this work—acknowledging all financial support and any other relevant financial or non-financial competing interests.<br><br>This statement is required for submission and **will appear in the published article** if the submission is accepted. Please make sure it is accurate and that any funding sources listed in your Funding Information	The authors have declared that no competing interests exist.

Powered by Editorial Manager® and ProduXion Manager® from Aries Systems Corporation

later in the submission form are also declared in your Financial Disclosure statement.<br><br>View published research articles from *PLOS ONE* for specific examples.<br><br>**NO authors have competing interests**<br><br>Enter: *The authors have declared that no competing interests exist.*<br><br>**Authors with competing interests**<br><br>Enter competing interest details beginning with this statement:<br><br>*I have read the journal's policy and the authors of this manuscript have the following competing interests: [insert competing interests here]*<br><br>* typeset	
**Ethics Statement**<br><br>Enter an ethics statement for this submission. This statement is required if the study involved:<br><br>• Human participants<br>• Human specimens or tissue<br>• Vertebrate animals or cephalopods<br>• Vertebrate embryos or tissues<br>• Field research<br><br>Write "N/A" if the submission does not require an ethics statement.<br><br>General guidance is provided below. Consult the submission guidelines for detailed instructions. **Make sure that all information entered here is included in the Methods section of the manuscript.**	The University of Michigan Institutional Review Board deemed this study Not Regulated (HUM00236811). Consent was not required, as only publicly available de-identified data was used.

**Format for specific study types**<br><br>**Human Subject Research (involving human participants and/or tissue)**<br>• Give the name of the institutional review board or ethics committee that approved the study<br>• Include the approval number and/or a statement indicating approval of this research<br>• Indicate the form of consent obtained (written/oral) or the reason that consent was not obtained (e.g. the data were analyzed anonymously)<br><br>**Animal Research (involving vertebrate animals, embryos or tissues)**<br>• Provide the name of the Institutional Animal Care and Use Committee (IACUC) or other relevant ethics board that reviewed the study protocol, and indicate whether they approved this research or granted a formal waiver of ethical approval<br>• Include an approval number if one was obtained<br>• If the study involved *non-human primates*, add *additional details* about animal welfare and steps taken to ameliorate suffering<br>• If anesthesia, euthanasia, or any kind of animal sacrifice is part of the study, include briefly which substances and/or methods were applied<br><br>**Field Research**<br><br>Include the following details if this study involves the collection of plant, animal, or other materials from a natural setting:<br>• Field permit number<br>• Name of the institution or relevant body that granted permission	
**Data Availability**<br><br>Authors are required to make all data underlying the findings described fully available, without restriction, and from the time of publication. PLOS allows rare exceptions to address legal and ethical concerns. See the PLOS Data Policy and FAQ for detailed information.	No - some restrictions will apply

Powered by Editorial Manager® and ProduXion Manager® from Aries Systems Corporation

A Data Availability Statement describing where the data can be found is required at submission. Your answers to this question constitute the Data Availability Statement and **will be published in the article**, if accepted.<br><br>**Important:** Stating 'data available on request from the author' is not sufficient. If your data are only available upon request, select 'No' for the first question and explain your exceptional situation in the text box.<br><br>Do the authors confirm that all data underlying the findings described in their manuscript are fully available without restriction?	
**Describe where the data may be found in full sentences. If you are copying our sample text, replace any instances of XXX with the appropriate details.**<br><br>• If the data are **held or will be held in a public repository**, include URLs, accession numbers or DOIs. If this information will only be available after acceptance, indicate this by ticking the box below. For example: *All XXX files are available from the XXX database (accession number(s) XXX, XXX.).*<br>• If the data are all contained **within the manuscript and/or Supporting Information files**, enter the following: *All relevant data are within the manuscript and its Supporting Information files.*<br>• If neither of these applies but you are able to provide **details of access elsewhere**, with or without limitations, please do so. For example:<br><br>*Data cannot be shared publicly because of [XXX]. Data are available from the XXX Institutional Data Access / Ethics Committee (contact via XXX) for researchers who meet the criteria for access to confidential data.*<br><br>*The data underlying the results presented in the study are available from (include the name of the third party*	The data underlying the results presented in the study are available from https://www.pmadata.org/data/request-access-datasets

and contact information or URL).<br>• This text is appropriate if the data are owned by a third party and authors do not have permission to share the data.<br><br>* typeset	
Additional data availability information:	Tick here if the URLs/accession numbers/DOIs will be available only after acceptance of the manuscript for publication so that we can ensure their inclusion before publication.

Powered by Editorial Manager® and ProduXion Manager® from Aries Systems Corporation

Manuscript

Click here to access/download;Manuscript;Manuscript.docx

Contraceptive use by number of living children in Ghan[illegible] evidence from the 2017 maternal health survey

Sarah D. Compton[1*], Emmanuel Nakua[2], Cheryl Moyer[3], Veronica Dzomeku[4], Emily Treleaven[5], Easmon Otupiri[6], Jody Lori[7]

[1]Program on Women's Healthcare Effectiveness Research, Department of Obstetrics and Gynecology, University of Michigan, Ann Arbor, Michigan, United States of America

[2]Department of Epidemiology and Biostatistics, School of Public Health, Kwame Nkrumah University of Science and Technology, Kumasi, Ghana

[3]Department of Learning Health Sciences and Department of Obstetrics and Gynecology, University of Michigan, Ann Arbor, Michigan, United States of America

[4]Department of Nursing and Midwifery, College of Health Sciences, Kwame Nkrumah University of Science and Technology, Kumasi, Ghana

[5]Survey Research Center, Institute for Social Research, University of Michigan, Ann Arbor, Michigan, United States of America

[6]Department of Population, Family, and Reproductive Health , Kwame Nkrumah University for Science and Technology, Kumasi, Ghana

[7]Global Affairs and Community Engagement, School of Nursing, University of Michigan, Ann Arbor, Michigan, United States of America

*Corresponding author
Email: sarahrom@med.umich.edu (SDC)

Abstract

Background

There is a significant literature describing the link between parity and contraceptive use. However, there is limited knowledge about the disaggregation by parity of the type of contraceptives. In this study, we describe the use of contraceptives by parity among women of reproductive age in Ghana, focusing on use of highly effective methods (injection, pill, intrauterine device, implant, and sterilization).

Methods

Using the 2017 Ghana Maternal Health Survey, a nationally-representative cross-sectional household survey, we describe contraceptive method use by number of living children among sexually active women of reproductive age. We then estimated predictors of use of highly effective contraception in a multilevel logistic regression model.

Results

Most women in this survey are not using any method of contraception, although this varies by whether or not they have begun childbearing. Contraceptive method use varies by number of living children. Before having children, natural (periodic abstinence and withdrawal) and episodic (condoms) methods dominate. Once a woman has one living child, method preference changes to injectables and implants. Factors associated with using a highly effective method of

contraception are: having >3 children, being in a relationship, having had an abortion, being younger than age 30, and having had sexual intercourse within days of answering the survey.

Conclusion

In this analysis, the number of living children a woman has, her age, and timing of last intercourse are the most significant predictors of using a highly effective method of contraception. However, the majority of participants in this study report not using any method of contraception to avoid unwanted pregnancies. Future research that attempts to unpack the disconnect between not wanting to become pregnant and not using contraception is warranted.

Background

Globally, many countries have experienced a significant decline in their fertility rates [1]. The decline in fertility has been slower across sub-Saharan African (SSA) countries compared to other regions of the world [2]. In Ghana, a small country in West Africa, studies have shown that not all women who desire to use family planning are able to freely access a desired method, while others have shown a stall in fertility decline [3, 4]. While use of contraception among Ghanaian women has increased since the mid-1980s, the rate has stalled at less than 25%. In 2006, the Maputo Programme of Action (MPoA), signed by 48 African heads of state, highlighted a need for universal accessibility to sexual and reproductive health (SRH) services [5]. The revised MPoA 2016-2030 provides a renewed and more robust commitment by African leaders to facilitate and provide the enabling environment for attainment of universal access to SRH services. Similarly, in 2012, 41 countries, including Ghana, ratified actions to ensure access to contraceptives [6]. Following these policies and programmes of action, the United Nations and its partners introduced the 17 Sustainable Development Goals (SDGs), of which Target 3.7 seeks to ensure universal access to SRH services, including family planning and contraceptives by the year 2030 [7]. Despite these actions, the use of contraceptives, particularly modern methods, remains low in SSA countries compared to other regions of the world.

Of the estimated 1.1 billion women of reproductive age globally who are currently sexually active, 24.5% report not wanting to become pregnant any time soon, indicating an unmet need for contraception [8]. Although low contraceptive use is a public health concern across the globe, it appears to be an endemic issue in SSA. A study of 17 SSA countries revealed that only 17% of reproductive-aged women use contraceptives [9]. In Ghana, the 2014

Demographic and Health Survey found modern contraceptive use to be low among women aged 15–19 (19%) and 45–49 (18%) [10]. However, in that same survey, more than 40% of respondents reported they do not want to have any more children [11].

A combination of socioeconomic, demographic, cultural, and contextual factors have been found to significantly predict the use of contraceptives. For example, socioeconomic factors such as household wealth, place of residence, and educational attainment have been identified as significant factors that influence women's use of contraceptives [11, 12]. Likewise, contextual and cultural issues such as misperceptions about contraceptives, fear of side effects, and level of community literacy play a critical role in influencing women's use of contraceptives [11, 13]. From a demographic perspective, there is compelling evidence to show that women's age and parity are significantly associated with their use and type of contraceptives [14, 15]. Behrman et al. report that in West Africa, nulliparous women had higher modern contraceptive use than parous women. However, in many contexts in SSA, including Ghana, contraceptive use is considered inappropriate until after a first birth because women are under pressure to have children soon after marriage [16]. In the case of Ghana, this is evident in a total fertility rate of 3.9 and a median age at first birth of 21.5 years [17].

Despite evidence of a significant association between parity and contraceptive use, there is limited knowledge about the disaggregation by parity of the type of contraceptives used. Thus, the question that remains is, "What is the distribution of contraceptive use by parity in Ghana?" This question remains unanswered in previous studies conducted in Ghana or other SSA countries [18, 19], reflecting a significant gap in the current understanding of parity in relation to women's use of contraceptives. The present study seeks to narrow this knowledge gap by investigating the

use of contraceptives by parity among women of reproductive age. The analysis disaggregates the various types of contraceptives by the number of living children the woman has to provide a deeper understanding of the dynamics at play.

Methods

Using the 2017 Ghana Maternal Health Survey (GMHS) [20], we assessed method of contraception used by the number of living children among respondants who reported they have ever had sexual intercourse. GMHS is a nationally representative cross-sectional household survey with a two-stage stratified random sample. Households were randomly sampled within each enumeration area that was selected as a primary sampling unit with probability proportionate to size. Within each selected household, all women of reproductive age (15-49 years) were eligible to participate. Data collection was implemented by the Ghana Statistical Service and the Ghana Health Service from June 15-October 12, 2017 and analysis was carried out from January-June 2023. All data were anonymized by the Ghana Health Service and Ghana Statistical Service. The authors did not have access to information that could identify participants before or after data collection. The University of Michigan Institutional Review Board deemed this study Not Regulated (HUM00236811). Consent was not required, as only publicly available deidentified data were used.

To define the sub-sample of women who have never been pregnant (n=2,984), we started with the 4,544 who state they have 0 living children and have ever had sex. We then excluded those who stated they were pregnant at the time of the survey (n=427). We next excluded nulliparous women who reported they had terminated a pregnancy (n=596), had a miscarriage

(n=334), or experienced a stillbirth (n=126). Finally, we excluded women who reported a child death (n=77). While not a perfect measure of women who have never been pregnant, we believe it closely approximates this population.

Age was originally collected continuously in years. We created seven categories (<18, 18-24, 25-29, 30-34, 35-39, 40-44, 45+). Similarly, age at first intercourse was categorized into five groups (<15, 15-16, 17-18, 19-20, 21+). Next, we included a categorical measure of time since last intercourse. Respondents reported whether their last intercourse was "days ago," "weeks ago," "months ago," and "years ago"; responses are categorized as "within the last week," "within 1-4 weeks," "within 1-12 months," or "more than one year." Lastly, we created a dichotomous variable indicating whether a woman is currently using "highly effective contraception," defined as intrauterine device (IUD), implant, injection, pill, or sterilization.

Descriptive statistics are reported with GMHS-defined survey weights. Via logistic regression with standard errors clustered by enumeration area, we analyzed factors associated with using a highly effective method of contraception among women who have had sex (n=21,397). Those factors associated with the outcome in bivariate analysis, and those we felt it important to control for a priori, were included in the multivariate analysis. Covariates included whether a woman is currently in a relationship; educational attainment (none, any primary, any junior secondary, any secondary, any post-secondary/higher); household wealth quintile; place of residence (urban, rural); whether the respondent has ever been pregnant; whether the respondent has ever had an abortion; number of living children; age; age at first intercourse; and time since last intercourse. Results are presented as odds ratios and marginal effects (generated using the *margins* command in Stata 16).

Results

A total of 25,062 women were included in the survey, and 21,397 in the regression. The majority of respondents (n=19,073, 76.1% full sample; n=15,429, 72.1% regression sample) were not using contraception. Table 1 displays the general demographics of our sample.

Table 1. Demographics of the study sample.

Variable	**0 children** N=8,209	**1+ children** N=16,853	**Full Sample** N=25,062	**Regression Sample** N=21,397
**Age, years**				
<18	3,863 (47.1)	300 (1.8)	4,163 (16.6)	1,348 (6.3)
18-24	2,957 (36.0)	3,040 (18.0)	5,997 (23.9)	5,236 (24.5)
25-29	778 (9.5)	3,335 (19.8)	4,113 (16.4)	4,043 (18.9)
30-34	289 (3.5)	3,270 (19.4)	3,559 (14.2)	3,545 (16.6)
35-39	159 (1.9)	2,927 (17.4)	3,086 (12.3)	3,083 (14.4)
40-44	108 (1.3)	2,290 (13.6)	2,398 (9.6)	2,396 (11.2)
45+	55 (0.7)	1,691 (10.0)	1,746 (7.0)	1,746 (8.2)
**Education**				
None	521 (6.4)	5,987 (35.5)	6,508 (26.0)	6,376 (29.8)
Primary	1,112 (13.5)	3,634 (21.6)	4,746 (18.9)	4,116 (19.2)
Junior Secondary	3,219 (39.2)	4,627 (27.5)	7,846 (31.3)	6,072 (28.4)
Senior Secondary	2,465 (30.0)	1,715 (10.2)	4,180 (16.7)	3,243 (15.2)
Post-Secondary/Higher	892 (10.9)	890 (5.3)	1,782 (7.1)	1,590 (7.4)
**Wealth quintile**				
Lowest	1,758 (21.4)	5,167 (30.7)	8,734 (26.5)	5,801 (27.1)
Second	1,349 (16.4)	3,365 (20.0)	7,104 (21.6)	4,657 (21.8)
Middle	1,486 (18.1)	2,961 (17.6)	6,183 (18.8)	4,037 (18.9)
Fourth	1,727 (21.0)	2,904 (17.2)	5,783 (17.6)	3,703 (12.3)
Highest	1,889 (23.0)	2,456 (14.6)	5,144 (15.6)	3,199 (15.0)
**Place of residence**				
Urban	4,701 (57.3)	7,843 (46.5)	12,544 (50.1)	10,605 (49.6)
Rural	3,508 (42.7)	9,010 (53.5)	12,518 (49.9)	10,792 (50.4)

**In a relationship**				
Yes	1,297 (15.8)	13,755 (81.6)	15,052 (60.1)	15,048 (70.3)
No	6,912 (84.2)	3,098 (18.4)	10,010 (39.9)	6,349 (29.7)
**Number of living children**				
0	–	–	8,209 (32.8)	4,544 (21.2)
1	–	–	3,951 (15.8)	3,951 (18.5)
2	–	–	3,456 (13.8)	3,456 (16.2)
3	–	–	3,021 (12.1)	3,021 (14.1)
4	–	–	2,474 (9.9)	2,474 (11.6)
5	–	–	1,741 (7.0)	1,741 (8.1)
6	–	–	1,118 (4.5)	1,118 (5.2)
7+	–	–	1,092 (4.4)	1,092 (5.1)
**Contraceptive method**				
Not using	7,150 (87.1)	11,923 (70.7)	19,073 (76.1)	15,429 (72.1)
Pill	152 (1.9)	652 (3.9)	804 (3.2)	804 (3.8)
Injection	123 (1.5)	1,662 (9.9)	1,785 (7.1)	1,783 (8.3)
Implant	56 (0.8)	1,261 (7.5)	1,317 (5.3)	1,316 (6.2)
IUD	3 (.04)	99 (.59)	102 (.41)	102 (.48)
Condom	236 (2.9)	148 (0.9)	384 (1.5)	381 (1.8)
Periodic abstinence	245 (3.0)	538 (3.2)	783 (3.1)	774 (3.6)
Withdrawal	78 (1.0)	143 (0.9)	221 (0.9)	220 (1.0)
Emergency contraception	142 (1.7)	115 (0.7)	257 (1.0)	257 (1.2)
Sterilization	4 (0.1)	223 (1.3)	227 (.91)	227 (1.1)
Other method	20 (4.8)	89 (0.5)	109 (.43)	104 (.49)
**Ever had sex**				
Yes	4,544 (55.4)	16,853 (100)	21,397 (85.4)	21,397 (100)
No	3,665 (44.6)	0	3,665 (14.6)	0
**Age at first sex, years**				
<15	415 (5.1)	2,260 (13.4)	2,675 (10.7)	2,675 (12.5)
15-16	1,091 (13.3)	4,810 (28.5)	5,901 (23.6)	5,901 (27.6)
17-18	1,386 (16.9)	5,170 (30.7)	6,556 (26.2)	6,556 (30.6)
19-20	952 (11.6)	2,979 (17.7)	3,931 (15.7)	3,931 (18.4)
21+	4,365 (53.2)	1,634 (9.7)	5,999 (23.9)	5,999 (10.9)
**Ever been pregnant**				
Yes	1,247 (15.2)	16,853 (100)	18,413 (73.5)	17,695 (82.7)
No	6,962 (84.8)	0 (0)	6,649 (26.5)	3,702 (17.3)

**Ever had an abortion**				
Yes	649 (7.9)	3,053 (18.1)	3,702 (14.8)	3,702 (14.8)
No	7,560 (92.1)	13,800 (81.9)	21,360 (85.2)	21,360 (85.2)
**Time since last intercourse**				
Within one week	1,052 (23.2)	5,307 (31.5)	6,359 (29.7)	6,359 (29.7)
Within 1-4 weeks	781 (17.2)	3,801 (22.6)	4,582 (21.4)	4,582 (21.4)
Within 1-12 months	1,850 (40.7)	5,425 (32.2)	7,275 (34.0)	7,275 (34.0)
More than one year	861 (18.9)	2,320 (13.8)	3,181 (14.9)	3,181 (14.9)

Data presented as n (%)

For those women who reported using contraception, method choice varied by the number of living children (Fig 1). Those with no living children rely most heavily on periodic abstinence and condoms (23.1% of all women with no children who use contraception use periodic abstinence while 22.1% use condoms), and the pill (14.4%). Injectables became the method of choice for women once they have one living child, although implants increased as a method of choice once a woman had 5 living children, and was the most popular method for those with 6 or more living children.

Fig 1. Method of contraception used by number of living children. LAM=lactation amenorrhea method; IUD=intrauterine device

Of the 2,984 women in the GMHS who have never been pregnant but reported ever having sex, 2,265 (75.9%) were not using a method of contraception. Among those who were using a method of contraception, condoms were most commonly used, followed by periodic abstinence, withdrawal, and the pill. Data not shown.

The results show that 28.5% of women who have been pregnant are currently using contraception, while only 11.2% of those who have not been pregnant are currently using contraception ($p<.001$) (Fig 2).

Fig 2. Rates of current contraception use by pregnancy and sex history.

In our regression analysis reflecting use of highly effective contraception, participants in a relationship are 2.6% less likely (marginal effect: -.026; p=.002) to be using highly effective contraception, while those who have had an abortion are 4.5% more likely. Compared with people with a primary education, those with no education are 4.7% less likely to use highly effective contraception andhigher education is associated with an increased likelihood of using highly effective contraception (2.8%, 2.3% 3.1% more likely for those who have attended Junior Secondary, Senior Secondary, and higher respectively).

Of interest, those with 0, 1, or 2 children, compared to those with 3, are less likely to be using highly effective contraception (19.4%, 12.0%, and 5.6%, respectively), while those with 4, 5, 6, and 7+ living children are more likely to be using highly effective contraception (4.5%, 8.3%, 11.8%, and 11.8%, respectively). Younger participants are more likely to be using highly effective contraception, compared to those aged 30-34. Participants older than age 35 are less likely to be using highly effective contraception. Participants who have had intercourse within days of the survey are 4.4% more likely to be using highly effective contraception than those who have not had intercourse in “weeks,” and those who have not had sex in months or years are 8.2% and 15.5% less likely, respectively.

The full results of our logistic regression can be seen in Table 2.

Table 2. Logistic regression illustrating factors associated with use of highly effective contraception.[a]

Variable	Coefficient	95% CI	p-value	Marginal Effect
**Age**				
<18	.222	-.014- .458	.065	.035
18-24	.592	.465-.719	<.001	.100
25-29	.327	.219-.434	<.001	.052
30-34	Ref			
35-39	-.215	-.332- -.099	<.001	-.030

40-44	-.472	-.612- -.332	<.001	-.062
45+	-.923	-1.11- -.742	<.001	-.106
**Education**				
None	-.337	-.449- -.222	<.001	-.047
Primary	Ref	Ref	ref	Ref
Junior Secondary	.176	.072-.280	.001	.028
Senior Secondary	.146	.012-.279	.032	.023
Post-Secondary/Higher	.193	.007- .378	.042	.031
**Wealth quintile**				
Lowest	.021	-.103-.144	.749	.003
Second	.053	-.060-.167	.356	.008
Middle	Ref			
Fourth	.011	-.104-.127	.851	.002
Highest	-.117	-.250- -.016	.084	-.017
**Place of residence**				
Urban	Ref	ref	ref	ref
Rural	.152	.052-.251	.003	.023
**In a relationship**	-.292	-.369- -.187	<.001	-.026
**Number of living children**				
0	-1.55	-1.74- -1.35	<.001	-.194
1	-.783	-.915- -.652	<.001	-.120
2	-.327	-.452- -.202	<.001	-.056
3	ref	Ref	ref	Ref
4	.229	.106-.371	<.001	.045
5	.428	.276-.579	<.001	.083
6	.590	.422-.758	<.001	.118
7+	.592	.400-.783	<.001	.118
**Age at first sex**				
<15	-.015	-.135-.105	.804	-.002
15-16	ref	ref	Ref	Ref
17-18	.017	-.076-.110	.719	.003
19-20	.019	-.084- .123	.714	.003
21+	-.108	-.253- .036	.142	-.016
**Ever been pregnant**	.080	-.126-.287	.446	.012
**Ever had an abortion**	.293	.197-.390	<.001	.045
**Time since last intercourse**				
Within one week	.244	.155-.334	<.001	.044
Within 1-4 weeks	ref	Ref	ref	Ref
Within 1-12 months	-.534	-.629- -.439	<.001	-.082
More than one year	-1.23	-1.39- -1.08	<.001	-.155

[a]IUD, implant, injection, pill, or sterilization

Discussion

The analysis in the present study disaggregates the method of contraception used by Ghanaian women by the number of living children they have. Of particular interest to this analysis, number of living children, age, and timing of last intercourse were the most significant predictors of using modern contraception. Specifically, participants with <3 living children were less likely to use highly effective contraception (16.9%, 11.6%, and 11.6% for 0, 1, and 2 children respectively), while those ≥4 living children were more likely (4.0%, 7.6%, 10.7%, and 10.6% for 4, 5, 6, and 7 children respectively). Participants older than age 45 and those who have not had sexual intercourse in "years" were 12.7% and 17.5% less likely to be using highly effective contraception.

Previous research on this topic has had contradictory outcomes. Our findings are contrary to a study conducted in the Democratic Republic of Congo that found parity not significantly associated with the use of modern contraception, and a study in Botswana [21] that found a negative association between parity and modern contraceptive use. However, our findings support a study in Zambia [22] and one in Malawi [23], where higher parity was associated with contraceptive use. However, the study from Zambia showed that women who ever had a pregnancy and those with a history of abortion were less likely to be using contraception, while our analysis showed the opposite: women who have ever been pregnant and those who have had an abortion are 7.5% and 3.9% more likely to be using highly effective contraception respectively.

Our findings are consistent with research conducted in Accra, Ghana [24], which found women with lower levels of education being less likely to use modern contraception. Further, we

found that higher education was associated with an increased likelihood of using highly effective contraception, as has been consistently found in previous work in Ghana [25, 26] and across SSA [27-29].

This study also shows that older persons of reproductive age are more likely to use highly effective contraception. The result is similar to research in Malawi [23] and Ethiopia [30], where older age was negatively associated with using contraception. Previous research [23, 25, 31] has demonstrated that increasing wealth is associated with higher odds of using contraception. Our analysis indicates a reduced likelihood of using highly effective contraception for women in the highest wealth quintile, and no association at other levels of wealth. In Ghana, the most commonly used contraceptives (implant and injection), as well as most other methods, are available free of charge at many places, so it is not surprising that wealth is not associated with being able to obtain contraceptives.

Similar to previous work conducted in Nigeria [32], the vast majority of our participants are not using any modern method of contraception. However, unlike the study from Nigeria, our analysis shows an increasing likelihood of using modern contraception as parity increases. Perhaps the positive association between parity and modern contraceptive use could be explained from the point that nulliparous women and those with lower parity may be afraid of triggering primary or secondary infertility due to their reproductive choices, including using modern contraceptives [33]. However, in the case of multiparous and grand multiparous women, this fear may be non-existent, which may inform their higher likelihood of using modern contraceptives. Our findings are also contradictory to the study from Nigeria in terms of maternal age; our participants were less likely to be using highly effective contraception as age increased.

In general, however, most of the women in this survey are not using any method of contraception, despite a strong family planning program and overall decreases in total fertility in the country. Qualitative work in Accra [34] has shown that many women use "periodic contraception," where they rely on periodic abstinence and using emergency contraception to avoid pregnancy if they have intercourse on days deemed unsafe, then rely on abortion if those methods fail. It is noted that these methods, especially fertility awareness methods, are under-counted in surveys such as Demographic and Health Surveys, which might explain our findings.

Women's choice of method changes with the number of living children they have. While periodic abstinence and condoms are the methods of choice for women with 0 living children, once they have children, women adopt injectable contraception and the implant at much higher rates—though still not at what would be considered 'high' rates. Sterilization, not surprisingly, becomes more common with higher numbers of living children. Previous work [34] has highlighted the importance of future fertility and regular menses for many women, which may explain why many women avoid hormonal contraception until after having one or more children.

While other studies [35] have found that women in relationships are more likely to use contraception, our findings show that women who say they are in a relationship—either married or living together—are 2.6% less likely to use a highly effective method of contraception. This supports more recent work in Ghana, which has shown the importance of natural family planning within relationships [34]. It is possible that women without living children want to use highly effective contraception but are limited by provider bias, where highly effective and long-term methods are only offered to parous women, as was true in a study in Tanzania [36].

The majority of participants in our study reported not using any method of contraception to avoid unwanted pregnancies. Future research that attempts to unpack the disconnect between not wanting to become pregnant and not using contraception is warranted.

This study is not without limitations. Using cross sectional data precludes our ability to make causal inferences. Further, the data are from 2017, and the situation may have changed since then. Due to the secondary nature of the data, we were limited in the analyses we were able to perform. Certain factors we would have controlled for, such as desired family size, were not in the survey, so we were not able to assess those. Finally, this analysis only investigated individual-level factors associated with the use of contraception. There are family and community factors not accounted for in this analysis that might be highly influential in a woman's decision to use or not use highly effective contraception.

Conclusion

This study investigated the association between number of living children and contraceptive use. In our analysis, having a low level of education, being in a relationship, being older, having <3 living children, not having had sexual intercourse in months or years, and being in the highest wealth quintile are negatively associated with using highly effective contraception. Conversely, living in a rural area, having a history of abortion, having >3 living children, being younger, being more educated, and having had sex "within days" of the survey were positively associated with using highly effective contraception. However, more than eight in 10 survey respondents are not using any method of contraception. Understanding some of the seemingly

contradictory findings, such as rural residence being negatively associated with using contraception, are deserving of further investigation.

References

1. Bongaarts J. Global fertility and population trends. Semin Reprod Med. 2015;33(1): 5-10.

2. Bongaarts J. Africa's Unique Fertility Transition. Popul Dev Rev. 2017;43(S1): 39-58.

3. Grimm M, Günther I, Harttgen K, Klasen S. Slowdowns of fertility decline. Demogr Res. 2022;46(26): 737-66.

4. Schoumaker B. Stalls in Fertility Transitions in sub-Saharan Africa: Revisiting the Evidence. Stud Fam Plann. 2019;50(3): 257-78.

5. Munyati BM. African women's sexual and reproductive health and rights: The revised Maputo Plan of Action pushes for upscaled delivery. Agenda. 2018;32(1): 36-45.

6. Ahmed S, Choi Y, Rimon JG, Alzouma S, Gichangi P, Guiella G, et al. Trends in contraceptive prevalence rates in sub-Saharan Africa since the 2012 London Summit on Family Planning: results from repeated cross-sectional surveys. Lancet Glob Health. 2019;7(7): e904-e11.

7. Stephenson R, Gonsalves L, Askew I, Say L, WHO Working Group for Operationalizing Sexual Health. Detangling and detailing sexual health in the SDG era. Lancet. 2017;390(10099): 1014-5.

8. Kantorova V, Wheldon MC, Ueffing P, Dasgupta ANZ. Estimating progress towards meeting women's contraceptive needs in 185 countries: A Bayesian hierarchical modelling study. PLoS Med. 2020;17(2): e1003026.

9. Ba DM, Ssentongo P, Agbese E, Kjerulff KH. Prevalence and predictors of contraceptive use among women of reproductive age in 17 sub-Saharan African countries: A large population-based study. Sex Reprod Healthc. 2019;21: 26-32.

10. Ghana Statistical Service (GSS), Ghana Health Service (GHS), ICF International. Ghana Demographic and Health Survey 2014. Rockville, Maryland, USA: GSS, GHS, and ICF International. 2014. Available from: https://dhsprogram.com/pubs/pdf/FR307/FR307.pdf.

11. Ahinkorah BO, Budu E, Aboagye RG, Agbaglo E, Arthur-Holmes F, Adu C, et al. Factors associated with modern contraceptive use among women with no fertility intention in sub-Saharan Africa: evidence from cross-sectional surveys of 29 countries. Contracept Reprod Med. 2021;6(1): 22.

12. Aviisah PA, Dery S, Atsu BK, Yawson A, Alotaibi RM, Rezk HR, et al. Modern contraceptive use among women of reproductive age in Ghana: analysis of the 2003-2014 Ghana Demographic and Health Surveys. BMC Womens Health. 2018;18(1): 141.

13. Muanda M, Gahungu Ndongo P, Taub LD, Bertrand JT. Barriers to Modern Contraceptive Use in Kinshasa, DRC. PLoS One. 2016;11(12): e0167560.

14. Behrman JA, Wright KQ, Grant MJ, Soler-Hampejsek E. Trends in Modern Contraceptive Use among Young Adult Women in sub-Saharan Africa 1990 to 2014. Stud Fam Plann. 2018;49(4): 319-44.

15. de Vargas Nunes Coll C, Ewerling F, Hellwig F, de Barros AJD. Contraception in adolescence: the influence of parity and marital status on contraceptive use in 73 low-and middle-income countries. Reprod Health. 2019;16(1): 21.

16. Bankole A, Malarcher S. Removing barriers to adolescents' access to contraceptive information and services. Stud Fam Plann. 2010;41(2): 117-24.

17. Ghana Statistical Service (GSS), Ghana Health Service (GHS), ICF. Ghana Malaria Indicator Survey 2016. 2017. Available from: https://dhsprogram.com/pubs/pdf/MIS26/MIS26.pdf.

18. Achana FS, Bawah AA, Jackson EF, Welaga P, Awine T, Asuo-Mante E, et al. Spatial and socio-demographic determinants of contraceptive use in the Upper East region of Ghana. Reprod Health. 2015;12: 29.

19. Eliason SK, Bockarie AS, Eliason C. Postpartum fertility behaviours and contraceptive use among women in rural Ghana. Contracept Reprod Med. 2018;3: 13.

20. Ghana Statistical Service (GSS), Ghana Health Service (GHS), ICF. Ghana Maternal Health Survey 2017. 2018. Available from: https://dhsprogram.com/pubs/pdf/FR340/FR340.pdf.

21. Nair PS, Navaneetham K. Patterns and Determinants of Modern Contraceptive Use in Southern Africa Demogr India. 2015;44(1&2): 40-9.

22. Lasong J, Zhang Y, Gebremedhin SA, Opoku S, Abaidoo CS, Mkandawire T, et al. Determinants of modern contraceptive use among married women of reproductive age: a cross-sectional study in rural Zambia. BMJ Open. 2020;10(3): e030980.

23. Adebowale SA, Adedini SA, Ibisomi LD, Palamuleni ME. Differential effect of wealth quintile on modern contraceptive use and fertility: evidence from Malawian women. BMC Womens Health. 2014;14(1): 40.

24. Adanu RM, Seffah JD, Hill AG, Darko R, Duda RB, Anarfi JK. Contraceptive use by women in Accra, Ghana: results from the 2003 Accra Women's Health Survey. Afr J Reprod Health. 2009;13(1): 123-33.

25. Apanga PA, Adam MA. Factors influencing the uptake of family planning services in the Talensi District, Ghana. Pan Afr Med J. 2015;20: 10.

26. Eliason S, Awoonor-Williams JK, Eliason C, Novignon J, Nonvignon J, Aikins M. Determinants of modern family planning use among women of reproductive age in the Nkwanta district of Ghana: a case-control study. Reprod Health. 2014;11(1): 65.

27. Asiimwe JB, Ndugga P, Mushomi J, Manyenye Ntozi JP. Factors associated with modern contraceptive use among young and older women in Uganda; a comparative analysis. BMC Public Health. 2014;14: 926.

28. Kidayi PL, Msuya S, Todd J, Mtuya CC, Mtuy T, Mahande MJ. Determinants of Modern Contraceptive Use among Women of Reproductive Age in Tanzania: Evidence from Tanzania Demographic and Health Survey Data. Advances in Sexual Medicine. 2015;5(3): 43-52.

29. Tekelab T, Melka AS, Wirtu D. Predictors of modern contraceptive methods use among married women of reproductive age groups in Western Ethiopia: a community based cross-sectional study. BMC Womens Health. 2015;15: 52.

30. Kaneda T, Bietsch K, Population Reference Bureau. 2015 World Population Data Sheet 2015. Available from: https://www.prb.org/wp-content/uploads/2015/12/2015-world-population-data-sheet_eng.pdf.

31. Rasooly MH, Ali MM, Brown NJ, Noormal B. Uptake and predictors of contraceptive use in Afghan women. BMC Womens Health. 2015;15: 9.

32. Solanke BL. Factors influencing contraceptive use and non-use among women of advanced reproductive age in Nigeria. J Health Popul Nutr. 2017;36(1): 1.

33. Boivin J, Carrier J, Zulu JM, Edwards D. A rapid scoping review of fear of infertility in Africa. Reprod Health. 2020;17(1): 142.

34. Machiyama K, Marston C, LaChance NT, Adda-Balinia T, Tapsoba P, Population Council. How are educated women in Ghana regulating fertility without high levels of modern contraceptive use? STEP UP Evidence Summary. 2018. Available from: https://knowledgecommons.popcouncil.org/cgi/viewcontent.cgi?article=1214&context=departments_sbsr-rh.

35. Ngome E, Odimegwu C. The social context of adolescent women's use of modern contraceptives in Zimbabwe: a multilevel analysis. Reprod Health. 2014;11: 64.

36. Sedekia Y, Jones C, Nathan R, Schellenberg J, Marchant T. Using contraceptives to delay first birth: a qualitative study of individual, community and health provider perceptions in southern Tanzania. BMC Public Health. 2017;17(1): 768.

Click here to access/download;Figure;Figure 1.tif

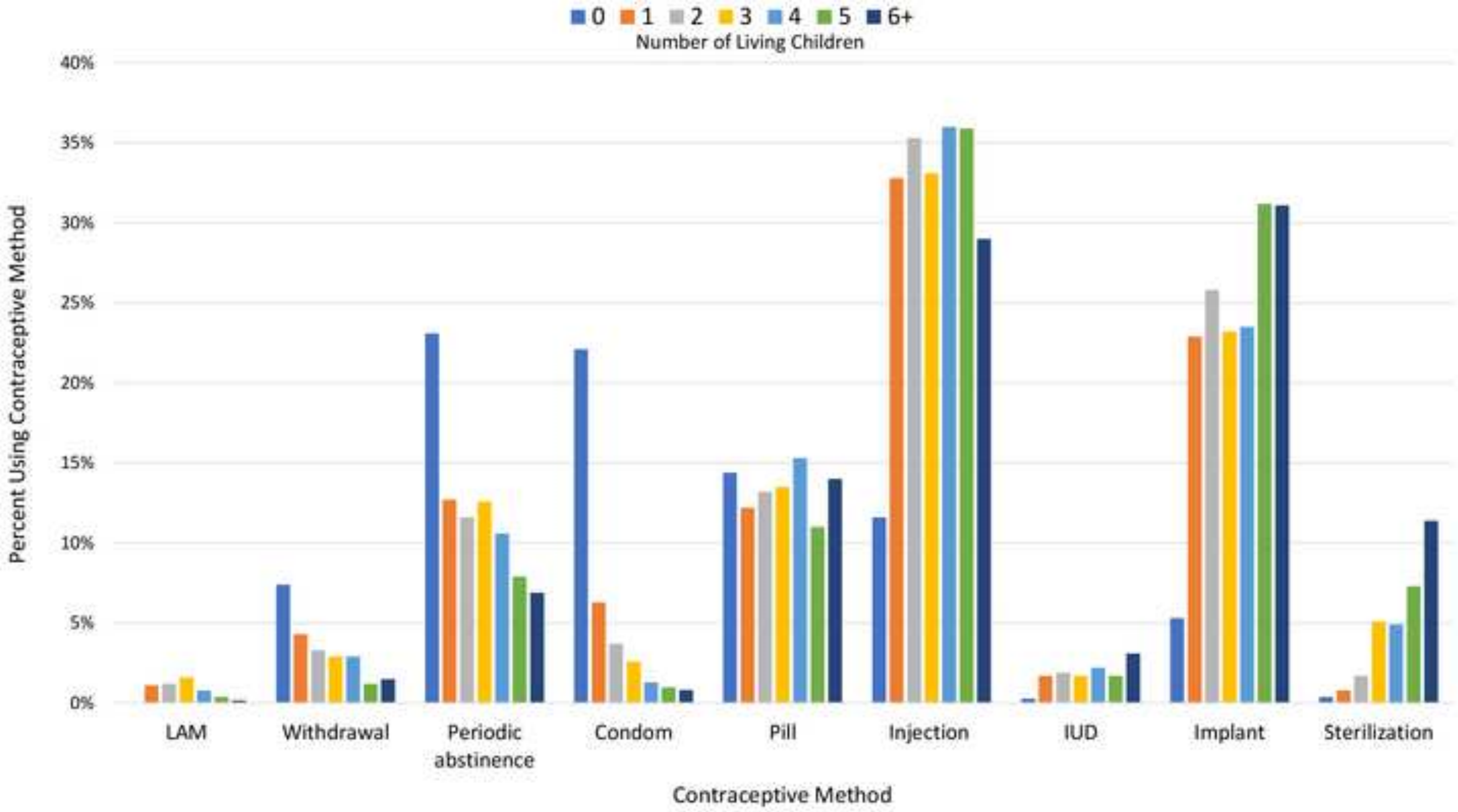

 Click here to access/download;Figure;Figure 2.tif

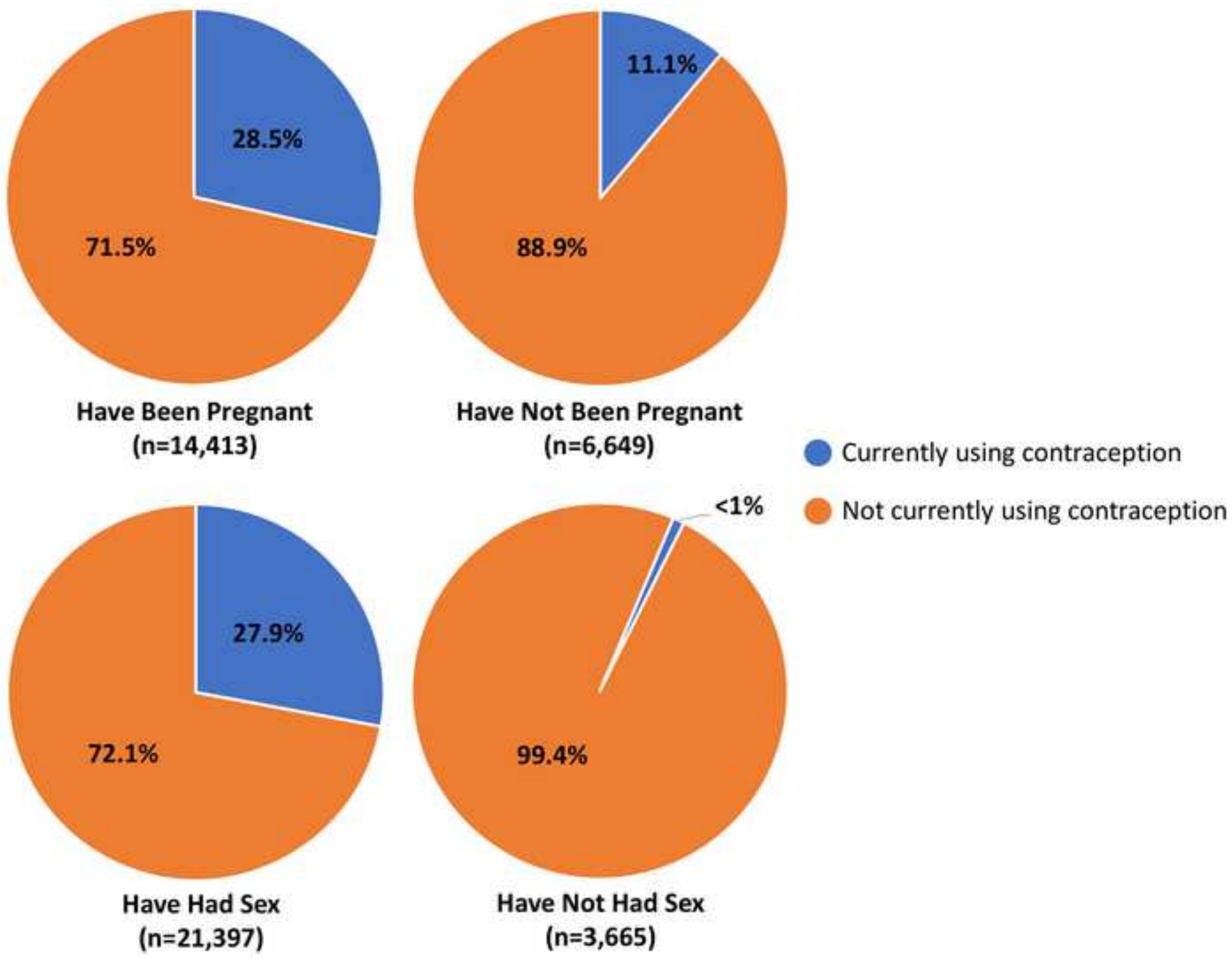

Supplement: Attachment — Submitted filename: PONE-D-23-19366_reviewer. DDL.pdf [file pone.0295815.s002.pdf]
