# Supplementary material for: Contraceptive use by number of living children in Ghana: Evidence from the 2017 maternal health survey
Source: PLoS One. 2023 Dec 14;18(12):e0295815. doi: 10.1371/journal.pone.0295815 (PMC10721096; doi:10.1371/journal.pone.0295815)
Supplement: Attachment — Submitted filename: Review_Contraceptive_15082023.docx [file pone.0295815.s001.docx]

*Date 15/08/2023*

*Dear editor,*

*Greetings*

*Thank you for asking me to review your paper titled “Contraceptive use by number of living children in Ghana: evidence from the 2017 maternal health survey”*

*General Concept*

*I think the paper is good but generally requires revision especially at the methods and results sections. It seeks to answer an important question of disaggregating parity and the use of contraceptives among women of childbearing age. Moreover, it can provide an important clue to the host country and policy makers on how contraceptives could be targeted among sexually active women by their number of living children. However, I have several concerns that need to be addressed. Here are a few comments to the authors.*

*Abstract*

*The strength of evidence using statistical significance (p values) must be indicated on page 3 lines 49-50. I recommend that the authors clearly define contraceptive use and coherently use this in all tables and figures.*

*Introduction*

*This part is very sound.*

*Methods*

*My significant concerns with the paper begin with the methods section followed by the results. The method section on statistical analysis does not indicate which method was used for the bivariate analysis such as Chi-square test of association between dichotomous variable and the demographic variables. Even though mentioned on page 7 line 138 that a bivariate analysis was performed, this result was never presented. The authors should include a bivariate analysis on table 2, as the results will help in understanding the final model selection criteria. Also, the final model presented using table 2 must be double checked as collinearity may exist between independent variables specifically (ever been pregnant, ever had an abortion and number of living children). After this check, the final model should be revised to exclude such variables for an accurate prediction of the adjusted model.*

*Results*

*This part needs a great improvement. All values in tables and figures must be checked.*

*The authors must carefully review and correct all errors on table 1 for the variables:*

*Ever had an abortion* *[3702+21360]* ***≠*** *regression sample total [21397]*

*Age at first sex years* *[2675+5901+6556+3931+5999]* ***≠*** *regression sample total [21397*

*Time since last intercourse* *[6359+4582+7275+3181]* ***≠*** *full sample total [25062]*

*Number presented on variable table 1 (Ever been pregnant [full model] [yes] =18413) and figure 2 (Have been pregnant, n= 14,413) are different and must be verified and corrected.*

*There are 8 categories (0,1..7+) for variable “Number of living children” presented on the two tables however the same variable has 7 categories (0,1..6+) presented at figure 1. The authors must ensure a consistent category is used throughout the manuscript.*

*Contraceptive method “LAM” is not shown on table 2 but presented at figure 1 whiles “Emergency contraceptives” is presented on table 2 but is not shown in figure 1.*

*I recommend that the authors consider including a mean/median age and standard deviation for the full sample and regression sample of respondents on contraceptive use on page 8 line 149. This will be useful in describing the demographic baseline characteristics of respondents. The updated table 2 should include an overall effect for each covariate as a supplement to the marginal/odds ratios provided for the bivariate and multivariate analysis.*

*Also ensure that you choose a consistent method of presenting ref/REF/Ref.*

*Discussion*

*The limitation section must be removed on page 16 line 261 as DHS data asks questions on fertility preferences, intentions, and desires- V602/5. Authors may add that the bias in self-reporting of total births may be misreported.*

*Conclusion*

*This part is relatively good though it can be more than this.*
